# Targeting *Loxosceles* spider Sphingomyelinase D with small-molecule inhibitors as a potential therapeutic approach for loxoscelism

**DOI:** 10.1080/14756366.2018.1546698

**Published:** 2019-01-07

**Authors:** Priscila Hess Lopes, Mário T. Murakami, Fernanda C. V. Portaro, Kerly Fernanda Mesquita Pasqualoto, Carmen van den Berg, Denise V. Tambourgi

**Affiliations:** a Immunochemistry Laboratory, Butantan Institute, São Paulo, SP, Brazil;; b Biosciences National Laboratory, National Centre for Research in Energy and Materials, Campinas, SP, Brazil;; c Alchemy – Innovation, Research & Development Ltda., Center of Innovation, Entrepreneurship and Technology (CIETEC), University of São Paulo, SP, Brazil;; d Centre for Medical Education, Cardiff University, School of Medicine, Cardiff, United Kingdom

**Keywords:** novel sphingomyelinase D inhibitors, *Loxosceles* venoms, molecular docking simulations, protein-ligand binding affinity, loxoscelism

## Abstract

*Loxosceles* spiders’ venoms consist of a mixture of proteins, including the sphingomyelinases D (SMases D), which are the main toxic components responsible for local and systemic effects in human envenomation. Herein, based on the structural information of SMase D from *Loxosceles laeta* spider venom and virtual docking-based screening approach, three benzene sulphonate compounds (named 1, 5 and 6) were identified as potential *Loxosceles* SMase D inhibitors. All compounds inhibited the hydrolysis of the sphingomyelin substrate by both recombinant and native SMases D. Compounds 5 and 6 acted as SMases D uncompetitive inhibitors with Ki values of 0.49 µM and 0.59 µM, respectively. Compound 1 is a mixed type inhibitor, and presented a Ki value of 0.54 µM. In addition, the three compounds inhibited the binding of SMases D to human erythrocytes and the removal of glycophorin C from the cell surface, which are important events in the complement-dependent haemolysis induced by *Loxosceles* venom. Moreover, compounds 5 and 6 reduced the binding of SMases to human keratinocytes membrane and the venom induced cell death. Importantly, compounds 5 and 6 also controlled the development of the necrotic lesion in an *in vivo* model of loxoscelism. Together, our findings indicate that the novel SMase D inhibitors presented here are able to suppress both local and systemic reactions induced by *Loxosceles* venoms. Since the number of *Loxosceles* envenomation accidents is currently growing worldwide, our results indicate that both inhibitors are promising scaffolds for the rational design of new drugs targeting SMases D from these spiders.

## 1. Introduction

Accidents with spiders from *Loxosceles* genus (Family Sicariidae) occur in temperate and tropical regions of North, Central and South Americas, Africa, Asia, Middle East, India, with new cases being reported recently, including fatal cases in Europe^1–9^.

Although the amount of venom inoculated during the bite by *Loxosceles* spiders does not exceed 30 μg of protein^10^, serious necrotic skin lesions can be observed and, less frequently, systemic effects may rise. Generally, the regression of necrotic lesions can take months and, regarding more severe cases, systemic effects can lead to death^11^.

Corticosteroid therapy and other drugs have been used in the treatment of loxoscelism, but without relevant outcomes or presenting undesirable side effects^12^. Although not available in the USA, the serum therapy for these spider bites is widely used in Brazil, even though there is no consensus regarding its efficacy, particularly on local lesions^13^.

Previously, we have demonstrated that the antiserum produced against sphingomyelinases D (SMases D; sphingomyelin phosphodiesterase D; E.C. 3.1.4.41) from *Loxosceles* spider venoms could efficiently neutralise the most toxic activities from homologous and heterologous *Loxosceles* venoms^14^. This and other studies have consolidated the fundamental role of SMases D as central components for the development of toxic effects induced by the *Loxosceles* venom^15–18^.

Our group has also solved the *Loxosceles* SMase D three-dimensional structure and its catalytic mechanism of sphingomyelin hydrolysis. The structure of the SMases D from spider venoms consists of a (β/α)_8_-barrel with a Mg^2+^ ion coordinated by acidic residues (Glu32, Asp34 and Asp91), which are relevant for substrate binding, and two catalytic histidine residues (His12 and His47)^19^^,^^20^. According to De Andrade et al.^20^ minor molecular modifications, such as amino acid substitutions on the surface loops, may affect the hydrolysis of sphingomyelin suggesting that, besides the active site, SMases D probably present motifs in its structure, which could participate in the molecular recognition process, binding affinity/mode, and hydrolysis of the substrate.

Although the molecular basis of sphingomyelin hydrolysis by SMases D was established, little is known about which region of these enzymes are responsible for the anchoring on cell surfaces, a central event on the mechanism of action of these toxins. In erythrocytes surface, the SMase D binding induces the activation of endogenous metalloproteases, which, in turn, cleave the sialic acid-rich extracellular portions of the glycophorins, rendering these cells activators of the complement alternative pathway, resulting in haemolysis^10^^,^^21^^,^^22^. Furthermore, the binding of SMase D to keratinocytes induces increased expression/secretion of matrix metalloproteinases (MMP-2, MMP-9 and MMP-7), which is possibly one of the main factors involved in the pathogenesis of the cutaneous loxoscelism^16^^,^^23^.

Thus, the investigation of small molecules capable of both abolishing SMases D hydrolytic activity and their ability to binding to cell surfaces seems to be a reasonable approach to develop effective SMases D inhibitors.

Considering the three dimension (3D) structural information, a virtual docking-based screening was performed in order to identify promising chemical compounds acting as potential inhibitors of the *Loxosceles* SMase D. The protein-ligand binding interactions were also investigated throughout molecular docking simulations to (*i*) map the complementary amino acid residues into, or near, the binding site, (*ii*) identify the type of molecular interactions established, and (*iii*) investigate the ligands’ binding mode and affinity. Using these approaches, we have selected three small benzene sulphonates synthetic compounds as promising inhibitors of SMase D from *Loxosceles* venom. In addition, we showed their properties on controlling important events associated with the loxoscelic envenomation, such as haemolysis and dermonecrosis.

## 2. Material and methods

### 2.1. Reagents, antibodies and buffers

*Reagents:* bovine serum albumin (BSA), paraformaldehyde, sphingomyelin (SM), choline oxidase, horseradish peroxidase (HRPO) and 3–(4-hydroxy-phenyl) propionic acid were purchased from Sigma Co. (St. Louis, MO, USA). 3–(4,5 dimethylthiazol-2yl)-2,5 diphenyltetrazolium bromide (MTT) was obtained from Invitrogen Corp. (Eugene, Oregon, USA). Dimethyl sulfoxide (DMSO) was obtained from Merck KGaA (Darmstadt, Germany) and BCA Protein Assay Kit from Pierce Biotechnology (Waltham, MA, USA). DMEM (Dulbecco’s Modified Eagle Medium) and penicillin-streptomycin were purchased from Gibco, Invitrogen Corp. (Eugene, Oregon, USA) and Foetal bovine serum (FBS) from Cultilab, (São Paulo, Brazil). *Antibodies:* Monoclonal antibody against GPC (Bric4, extracellular epitope aa 16 – 23) was obtained from IBGRL (Bristol, UK). Rabbit anti-mouse IgG-FITC (RAM-FITC) and rabbit anti-horse IgG-FITC (RAH-FITC) were obtained from Sigma Aldrich (St. Louis, MO, USA). Horse serum against recombinant SMases D from *L. intermedia* and *L. laeta* venoms were prepared as described^14^. *Buffers and solutions****:*** Alsever´s old solution: 114 mM sodium citrate, 27 mM glucose, 72 mM NaCl, pH 6.1. Phosphate Buffered Saline (PBS: 8.1 mM Na_2_HPO_4_; 1.5 mM KH_2_PO_4_; 137 mM NaCl; 2.7 mM KCl, pH 7.4). HEPES-buffered saline (HBS: NaCl 140 mM, KCl 5 mM, CaCl_2_ 1 mM, MgCl_2_ 1 mM, Hepes 10 mM, pH 7.4). FACS buffer (PBS buffer containing 1% of albumin and 0.1% of sodium azide). VBS^2+^ (2.8 mM barbituric acid, 145.5 mM NaCl, 0.8 mM MgCl_2_, 0.3 mM CaCl_2_, 0.9 mM Na-barbital, pH 7.2). Lowry reagents: CuSO_4_ 2%, Na_2_CO_3_ 2% prepared in NaOH 0.1 N, KNaC_4_H_4_O_6_ 4%, Folin Ciocalteau 1:2.

### 2.2. Spiders and venoms

Adult females of *L. laeta* spiders were provided by the Immunochemistry Laboratory, Butantan Institute, Brazil (capture and maintenance licences from IBAMA, Brazil, number 45166–6). The venoms were obtained by electrostimulation by the method of Bucherl^24^ with slight modifications. Briefly, 15–20 V electrical stimuli were repeatedly applied to the spider sternum and the venom drops were collected with a micropipette in sterile PBS, aliquoted and stored at –20 °C. The protein content of the venom samples was evaluated using the BCA Protein Assay Kit. Authorization of access to genetic resources process n° 02001.008541/2011–52, authorization 01/2009.

### 2.3. Recombinant Sphingomyelinase D

The recombinant Sphingomyelinase D (SMase D) from *L. laeta* venom was obtained as described by Fernandes-Pedrosa et al^25^. The protein content was determined using the method of Lowry et al^26^. The samples were stored at –80 °C until use.

### 2.4. Selection of the chemical compounds by virtual docking-based screening

The virtual docking-based screening was performed using the ZINC database^27^^,^^28^, which is a free database of commercially-available compounds (small molecules). The coordinates of SMase D from *L. laeta* venom were retrieved from the Protein Data Bank^29^, PBD ID 2F9R (resolution at 1.85 Å) (released in 6/27/2006; Murakami et al.^19^) and used as target reference to perform the molecular docking simulations, employing GOLD (Genetic Optimization for Ligand Docking) based on an genetic algorithm programme^30^.

The amino acid residues in the 3 D structure of SMase D considered to carry out molecular docking simulations were the following: His12, Tyr46, His47, Gly48, Thr49, Pro50, Cys51, Asp52, Val89, Asp91, Leu92, Lys93, Gly95, Ser132, Leu133, Pro134, Ser166, Asp164, Tyr169, Ser195, Gly197, Leu198, Tyr228, Trp230, Ser231, Met250. More than one hundred thousand compounds were virtually screened. The GoldScore fitness function^30^^,^^31^, which is the original scoring function provided with GOLD, was employed as a final criterion to classify the top 50 compounds. Furthermore, the compounds were pre-filtered in terms of their “drug-likeness” properties, by using “Lipinski’s rule of five”^32–34^. Thus, as result, a list of 14 compounds were identified as potential inhibitors of SMase D from *Loxosceles* venom, which were finally purchased from Aurora Fine Chemicals LLC (CA, USA).

### 2.5. Inhibition of the SMases D hydrolytic activity

The residual hydrolytic activity, after incubation of the SMase D with the chemical compounds, was estimated by determining the choline liberated from lipid substrates, using a fluorimetric assay^35^. Briefly, samples of SMase D (5 μg) were pre-treated or not (30 min, room temperature) with increased amounts of the chemical compounds (0 to 150 μg) solubilised in DMSO. Then, sphingomyelin (68.4 μM - final concentration), diluted in 1 ml of HEPES-buffered saline, was added and the reactions were developed for 30 min at 37 °C. After this incubation period, it was added a mixture consisting of 1 unit choline oxidase/mL, 0.06 units of horseradish peroxidase/mL, and 50 μM of 3–(4-hydroxy-phenyl) propionic acid in HBS, and the samples were incubated for 10 min at 37 °C. The choline liberated was oxidised to betaine and H_2_O_2_ and this product determined fluorimetrically at λem = 405 nm and λex = 320 nm, using 96-well microtiter plates, in a spectrofluorimeter (Victor 3^TM^, Perkin-Elmer, USA). Only the compounds able to produce an inhibitory effect higher than 20% upon the recombinant SMase D were selected. In addition, the selected compounds (20 μg) were further tested on the ability to reduce the SMase D activity presented in the whole *Loxosceles* venom (10 μg) as above described.

### 2.6. Determination of the hydrolysis inhibition mechanism and inhibition constant (*K*_i_) of the most active compounds

Fluorimetric experiments were performed, as above-mentioned, using four different concentrations of the sphingomyelin (SM) substrate, ranging from 0.058 to 0.23 µM. The reaction time was 15 min, the enzyme concentration set as 0.5 μg, and the most active inhibitors, identified in the various biological assays here performed, were tested at concentrations of 0.125 and 0.250 μM. For evaluating the inhibition mechanism, plots were constructed according to the Lineweaver-Burk method^36^ and the constants of inhibition (*K*_i_ values) calculated as described by Nagase and Salvensen^37^.

### 2.7. Exploring protein-ligands binding properties

After selection of the more efficient inhibitors (compounds 1,5 and 6) from the 14 chemical library, the exploitation of their binding modes into the *L. laeta* SMase D was carried out to verify the ligands’ accommodation into the binding site, as well as to identify the complementary amino acid residues and the molecular interactions established in the protein-ligands binding process.

The coordinates of the SMase D from *L. laeta* venom (PBD ID 2F9R; Murakami et al.^19^) were used to perform the molecular docking simulations of compounds **1**, **5** and **6**, employing CLC Drug Discovery Workbench 2.4 software (QIAGEN Aarhus A/S, 2014). The binding site was set up using two pockets to guide the molecular docking (30.21 Å^3^ and 14.34 Å^3^), which were near the catalytic region in 2F9R: chain A (295.42 Å^3^). The cut-off distance considered from the amino acid residues of binding site was 13 Å radius. The protonation state was included to the following amino acid residues: Asp135, Asp164, Asp177, Asp206 and Glu212. The calculation criteria to virtually screen the ligands are listed as follows: 1,000 iterations; 100% of top included docking results were kept; five docking results for each ligand were displayed (meaning five conformations for each ligand were considered); none water molecule was included. The score by PLANTS_PLP_ method^38^ was considered as evaluation criteria, and the binding mode of each ligand in the protein’s binding pocket was related to a score value (calculated binding affinity).

Herein, the score mimics the potential energy change when the target protein and ligand come together, meaning that a very negative score corresponds to a strong binding whereas a less negative, or even positive, score value corresponds to a weak or non-existing binding affinity. The total score value comprises the following types of contribution: hydrogen bond score, metal interaction score, steric interaction score, and ligand conformation penalty score. The ligand conformation penalty contribution scores the complementarity between the binding site and ligand by rewarding and punishing different types of heavy atom contacts having inter-atom distance less than 5.5 Å (CLC Drug Discovery Workbench Software®, version 2.4. QIAGEN Aarhus A/S, 2014). Thus, that contribution punishes internal heavy atom clashes in the ligand and strain resulting from unfavourable bond rotations. As the total score is a sum of contributions (score =  *S*_target-ligand_ + *S*_ligand_), a better score can be given to a large ligand, for instance, simply due to its size. In this regard, when comparing scores for different compounds, this effect must be considered. Furthermore, a set of docking results for each compound (more than one conformation for each ligand) should be performed to improve the chances of finding a pose having reduced ligand conformation penalty score. The molecular volume (V) of each compound considering the van der Waals radii was also calculated employing Discovery Studio Visualizer 4.0 software (Discovery Studio Visualizer, Accelrys Software Inc., 2005–2013).

### 2.8. Analysis of the inhibitors’ effects on the mechanism of haemolysis induced by *Loxosceles* venom

#### 2.8.1. Normal human serum and erythrocytes

Blood samples were collected without anticoagulant and allowed to clot for 4 h at 4 °C. After centrifugation, normal human serum (NHS) was collected and stored at –80 °C. Blood samples drawn to obtain erythrocytes (E), for subsequent use as target cells, were collected in anticoagulant (Alsever´s old solution). Human blood was obtained from healthy donors who knew the objectives of the study and signed the corresponding informed consent form, which was approved by the ethics committee (CAAE: 02212612.0.0000.5467).

#### 2.8.2. Treatment of erythrocytes with *Loxosceles* venom

Human erythrocytes were washed and resuspended at 1.5% in VBS^2+^ and incubated with venom (2.5 µg) in presence or absence of the inhibitory molecules (20 µg) for 30 min at 37 °C. Control samples were incubated with VBS^2+^. The cells were washed, resuspended to the original volume in VBS^2+^ and prepared for flow cytometry.

#### 2.8.3. Flow cytometry

Samples of human erythrocytes (25 μL of 1.5%) were incubated for 30 min with 25 µL of the primary antibodies (mouse MoAb anti-GPC, diluted 1:250, or horse serum anti-SMases D from *Loxosceles*, diluted 1: 500) in FACS buffer. After washing, cells were incubated with the appropriate FITC-labeled secondary antibodies (anti-mouse or anti-horse FITC in a dilution of 1:50) for 30 min. The cells were washed and fixed in FACS buffer containing 1% paraformaldehyde and analysed by flow cytometry (FACScalibur, Becton Dickinson, California, USA).

### 2.9. Analysis of the inhibitors effect on the cell viability and binding of SMases D to human keratinocytes membrane

#### 2.9.1. Cell culture

Human keratinocytes of HaCaT lineage, obtained from the Cell Bank of Rio de Janeiro (BCRJ - Rio de Janeiro, RJ, Brasil), were grown in 75 cm^2^ bottles (Corning Inc. - New York, USA) containing DMEM (Dulbecco’s Modified Eagle Medium) supplemented with 10% foetal bovine serum (FBS) and 1% penicillin-streptomycin, at 37 °C and 5% CO_2_.

#### 2.9.2. Venom treatment of the keratinocytes culture

HaCaT cells were subcultured in 96-well plates (5 × 10^4^ cells/well). Cells at 70–80% confluence were maintained overnight in DMEM without FBS, followed by incubation with the venom (10 µg of protein/well, respectively), in presence or absence of the chemical compounds (5 µg). DMEM, without FBS, was used as negative control. After 72 h, the viability of the cultures was tested by the MTT method^39^. The absorbance was measured in a spectrophotometer (Multiskan-EX, Labsystems, Helsinki, Finland) at 540 and 620 nm. The relative cell viability was calculated as: (Sample OD_(540–620 nm)_ – Background control OD_(540–620 nm)_)/(Control OD_(540–620 nm)_ – Background OD _(540–620 nm)_) × 100.

In order to analyse the effect of the inhibitors in SMase D ability to bind human keratinocytes membrane, samples of the cells (1 × 10^6^ cells/sample) were incubated with venom (10 μg) in the presence or absence of the inhibitors (20 μg), for 2 h at 37 °C, under agitation. Samples treated only with buffer or venom were used as negative and positive controls, respectively. After treatment, the cells were washed, labelled with specific antibodies, and analysed by flow cytometry regarding the presence of SMase D on the human keratinocytes membrane.

### 2.10. MMPs dosage in the culture supernatants from human keratinocytes

Supernatants of the keratinocyte cultures, treated for 72 h with venom in the presence or absence of inhibitors (5 μg), were centrifuged at 405*g* for 10 min, and tested for the presence of MMP-2 and MMP-9, using the MMP-2 ELISA Kit (QIA63-1EA) and MMP-9 ELISA Kit (QIA56-1EA), according to the manufacturer’s instructions (Calbiochem®, USA). The concentration of MMPs in the samples was calculated based on the standard curves of recombinant MMP-2 (390 to 50.000 pg/mL) and recombinant MMP-9 (78 to 20.000 pg/mL).

### 2.11. Inhibitory effects of the chemical compounds on the dermonecrosis induced by *Loxosceles* venom in vivo

#### 2.11.1. Animals

The synthetic compounds’ inhibitory effect on the development of the dermonecrotic lesion, induced by the *Loxosceles* venom, was investigated using albino New Zealand male rabbits weighing 3 kg. The animals were supplied by the Central Animal House of the Butantan Institute. Animals were maintained and used under strict ethical conditions according to the International Animal Welfare Organization. This study was submitted to the Institutional Animal Care Committee of the Butantan Institute (CEUAIB) and was approved by protocol # 805/11.

#### 2.11.2. Envenomation

Groups of adult albino rabbits (*n* = 12), weighing 3 kg, were inoculated intradermally on the shaved back with 5 μg/200 μL of *L. laeta* venom incubated with 1% DMSO in saline, or with inhibitors **1**, **5** and **6**, at concentration of 1 µg. As controls, the animals received injections containing sterile saline, DMSO, or only inhibitors. The dermonecrotic lesions were monitored and measured for up to 72 h. After this period, the animals were euthanized, the skin removed, fixed in 10% formalin pH 7.4, and the samples were submitted to histological analysis by staining with hematoxylin-Eosin. A dose-response curve was also performed using various concentrations of the inhibitors **5** (0.5; 1.0 and 1.5 µg) and **6** (0.7; 1.4 and 2.0 µg).

## 3. Results

### 3.1. Virtual docking-based screening and selection of more promising SMase D inhibitors

**Figure 1.**
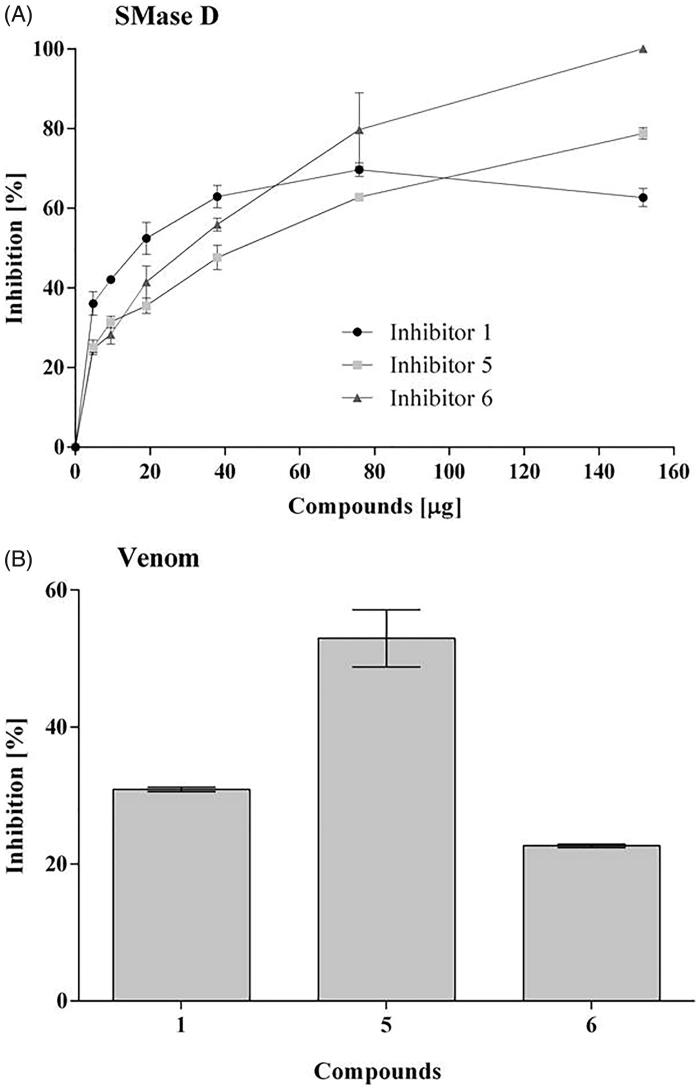
Action of the inhibitors on the hydrolysis of sphingomyelin by sphingomyelinase D. Samples of sphingomyelin (68 μM) were incubated for 30 min at 37° C with 5 μg of SMase D, previously incubated or not with 5 to 150 μg (5.8 × 10^−5^ to 9.2 × 10^−4^ M) of each inhibitor. Alternatively, samples of sphingomyelin with 10 μg of *Loxosceles* venom, previously incubated or not with 20 μg of each inhibitor. The release of choline, with consequent oxidation to betaine and H_2_O_2_, was determined in a fluorimeter and the hydrolysis expressed in arbitrary units of fluorescence. (**A**) Inhibition of the recombinant SMase D activity. (**B**) Inhibition of the sphingomyelinase activity of *L. laeta* venom. Data are expressed as percent inhibition of activity and they are represented as mean ± SEM of duplicates from three independent experiments. The graphs were built using GraFit software version 5.0.6.

Based on the generated scores in the virtual docking-based screening approach, the top fourteen compounds were selected for further analysis. These compounds presented similar GoldScore values, ranging from 99.28 to 87.38 (Table 1; Supplementary Figure S1). All of them were tested *in vitro* for the ability to inhibit the recombinant SMase D activity on the substrate sphingomyelin, but only compounds **1**, **5** and **6**, were able to significantly inhibit (higher than 20%) and in a dose dependent way the SMase D hydrolytic activity on SM (Figure 1A). The selected inhibitors belong to the benzene sulphonate class of organic compounds and they were named as 1 (6-amino-2-((4-cyanobenzyl)thio)pyrimidin-4-yl 4-methylbenzenesulphonate), 5 (4-bromo-N-[(E)-(2-methyl-1H-indol-3-yl)methyleneamino]benzenesulfonamide) and 6 (4-methyl-3-oxo-2–(3-pyridylmethylene)benzo[3,4-b]furan-6-yl 4-chlorobenzenesulphonate) (Table 2). Compounds 1, 5 and 6 were also able to inhibit the SMase D activity present in the whole *Loxosceles* venom, being compound 5 the most active (Figure 1B).

**Table 1.** Goldscore values presented by the fourteen compounds tested.

Compounds	Goldscore value	Compounds	Goldscore value
**1**	99.28	**8**	89.13
**2**	90.24	**9**	88.65
**3**	89.99	**10**	88.18
**4**	89.90	**11**	88.04
**5**	89.82	**12**	87.83
**6**	89.76	**13**	87.52
**7**	89.44	**14**	87.38

**Table 2.** Virtual docking-based screening findings for the three more promising *Loxosceles* SMase D inhibitors (five conformations of each compound), using CLC Drug Discovery Workbench software 2.4 (QIAGEN Aarhus A/S, 2014). 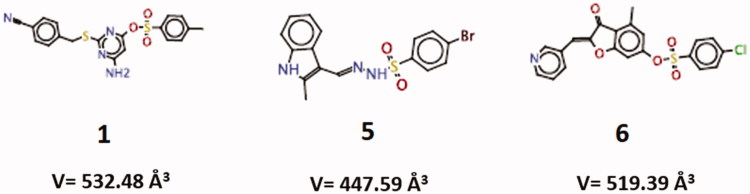

Compound	**Total score (kcal.mol**^–^**^1^)**	Flexible bonds	**Hydrogen bonding score (kcal.mol**^–^**^1^)**	**Steric interaction score (kcal.mol**^–^**^1^)**	**Ligand conformation penalty (kcal.mol**^–^**^1^)**
**1**	–74.80	7	–10.99	–68.32	4.51
**1**	–**74.40**	**7**	–**11.45**	–**67.32**	**4.38**
**1**	–74.27	7	–11.04	–67.63	4.40
**1**	–67.70	7	–11.90	–61.16	5.36
**1**	–67.10	7	–17.73	–54.36	5.00
**5**	–54.18	4	–7.24	–50.14	3.20
**5**	–53.88	4	–7.31	–49.36	2.79
**5**	–53.30	4	–6.64	–48.89	2.23
**5**	–52.19	4	–7.21	–46.41	1.43
**5**	–**51.65**	**4**	–**2.00**	–**50.45**	**0.80**
**6**	–55.31	4	–2.00	–61.23	7.93
**6**	–52.74	4	–2.00	–58.36	7.62
**6**	–49.05	4	–2.00	–54.30	7.25
**6**	–**48.23**	**4**	–**7.73**	–**46.68**	**6.18**
**6**	–47.22	4	–4.00	–49.99	6.77

Metal interaction score = 0 to all compounds.

### 3.2. Hydrolysis inhibition mechanism and protein-ligands binding analysis

**Figure 2.**
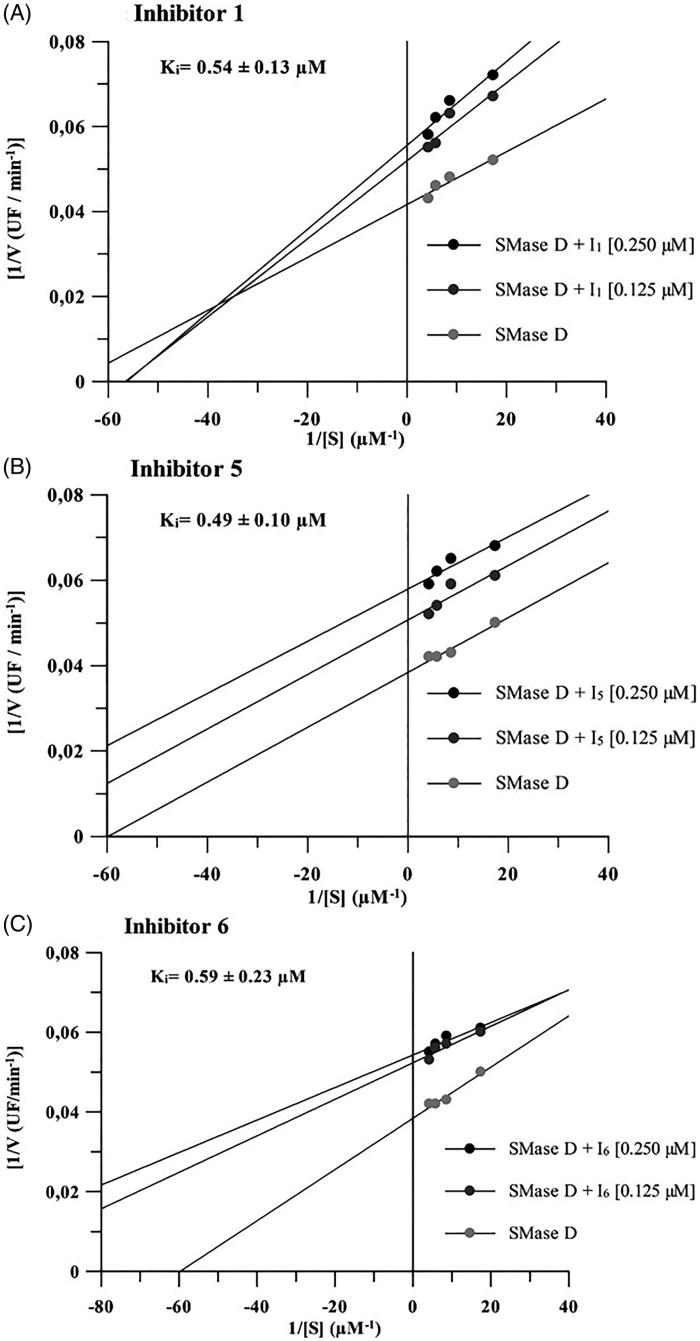
Mechanism of inhibition of compounds 1, 5 and 6 on the hydrolytic activity of SMase D on the sphingomyelin substrate. In these assays, four substrate concentrations (0.058 to 0.230 μM) were used, the enzyme concentration was kept fixed at 0.5 μg and the inhibitors were used at concentrations of 0.125 and 0.250 μM. The release of choline, with consequent oxidation to betaine and H_2_O_2_, was determined in a fluorimeter and the hydrolysis expressed in arbitrary fluorescence units as in the sphingomyelin hydrolysis assay. Data are expressed as mean ± standard error of triplicates representative of three independent experiments. For analysis, the Linewarver-burk plots method was used and the plots were build using GraFit software version 5.0.6.

The constants of inhibition (K*_i_* values) of the three compounds were calculated as described^37^. Similar K*i* values were observed, i.e. 0.54 ± 0.13 μM for compound 1, 0.49 ± 0.10 μM for compound 5 and 0.59 ± 0.23 μM for compound 6. Moreover, it was determined, by Lineweaver-Burk method^36^, that compound 1 exhibits different inhibition mechanism from those established to inhibitors 5 and 6. The inhibitor 1 exerts a mixed inhibition behaviour upon the sphingomyelin hydrolysis, while compounds 5 and 6 showed to be uncompetitive inhibitors regarding the SMase D hydrolytic activity (Figure 2).

These compounds were also analysed regarding their binding modes into the *L. laeta* SMase D^19^, using CLC Drug Discovery Workbench software 2.4 (QIAGEN Aarhus A/S, 2014). The five docking results for each compound are listed in Table 2. The conformation of each ligand, presenting lower energy value for the ligand conformation penalty contribution, was considered to further investigate the molecular interactions established with the SMase D structure. The total score values found for the compounds **1** (*V* = 532.48 Å^3^), **5** (*V* = 447.59 Å^3^) and **6** (519.39 Å^3^), were –74.40, –51.65 and –48.23 kcal/mol, respectively. The largest ligand (compound **1**) had better total score value (more negative). The difference between the total score values from compounds **5** and **6** was less than 5 kcal/mol indicating they may share similar binding affinities. These data positively correlate with the inhibition mechanism results, which show that compounds with the most similar score value (5 and 6) have the same mechanism of inhibition. Compound 1, however, has the most diverse score value and a different inhibition mechanism.

Furthermore, regarding the findings of the molecular docking simulations, the compounds **1**, **5** and **6**, have established, respectively, six, one and three hydrogen bonding interactions into the SMase D binding site (Table 3). Hydrogen bonding interactions involving the residue Tyr169 were found for the three compounds. However, only compound **1** established hydrogen bonding interactions with residues involved in the ion-metal coordination (Asp91). The list of complementary amino acid residues into the SMase D binding site that interact with each inhibitor is presented in Table 3.

**Table 3.** List of the complementary amino acid residues which establish interactions with the three more promising inhibitors into the *Loxosceles* SMase D binding site, according to the findings from molecular docking simulations (CLC Drug Discovery Workbench Software 2.4 (QIAGEN Aarhus A/S, 2014).

Compound 1	Compound 5	Compound 6
**Asp91 (1)**	**Tyr169 (1)**	**Lys93 (1)**
**Ser132 (1)**	Pro134	**Tyr169 (1)**
**Tyr169 (2)**	Ser166	**Asn200 (1)**
**Ser166 (1)**	Gly167	His47
**Ser195 (1)**	Pro168	Pro134
Val89	Leu170	Asp135
Lys93	Leu173	Ser166
Leu133	Leu198	Gly167
Pro134	Thr199	Pro168
Asp135	Asn200	Leu170
Asp164	Phe201	Leu173
Gly167	Asp206	Leu198
Pro168		Thr199
Leu170		Phe201
Leu173		Ser202
Pro174		
Asp196		
Gly197		
Leu198		
Thr199		
Asp206		
Asp209		
Tyr228		
Trp230		

The complementary amino acid residues in the SMase D binding site that establish hydrogen bonding interactions are in bold letters followed by the number of H bonds established between parentheses.

**Figure 3.**
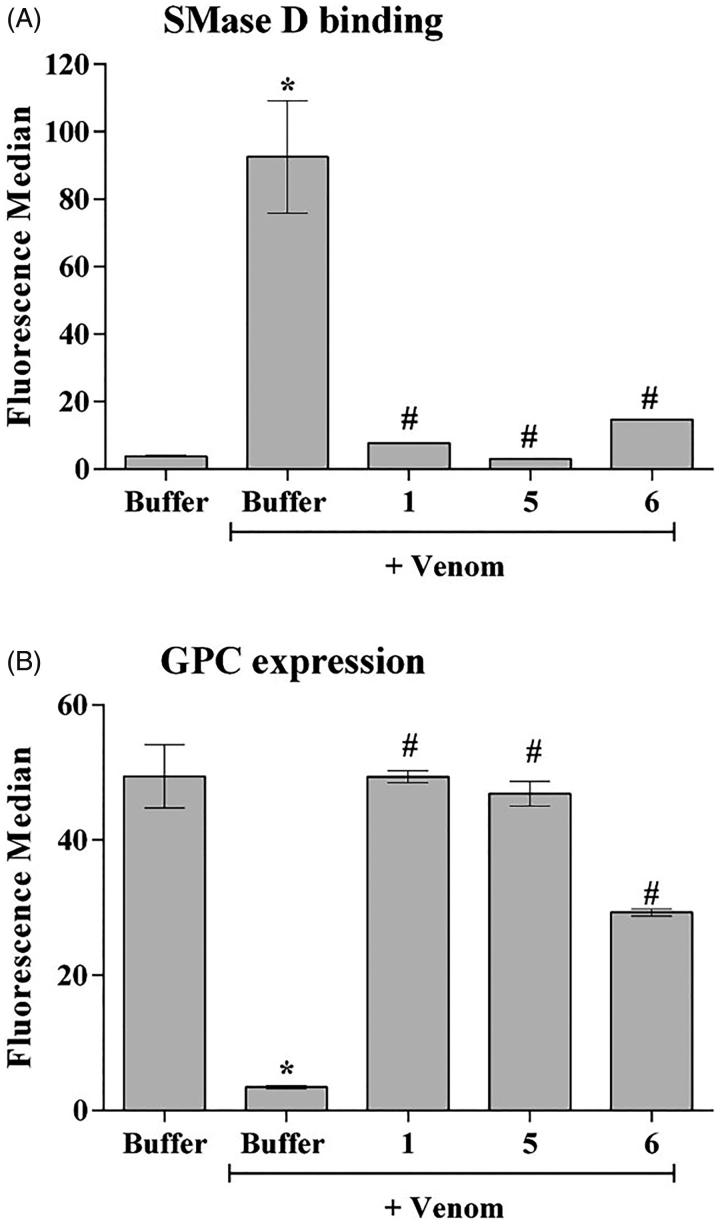
The ability of the compounds to inhibit the binding of SMases D to the surface of erythrocytes and to induce the cleavage of glycophorin C (GPC). Human erythrocytes were treated with 2.5 μg of the *L. laeta* venom incubated or not with 20 μg of each inhibitor and analysed for the expression of GPC by flow cytometry. The ability of the toxins to bind to the surface of the erythrocytes was analysed using a rabbit monospecific polyclonal serum against *L. intermedia* SMases D. **(A)** Binding of SMase D on the surface of human erythrocytes. **(B)** Removal of GPC induced by *L. laeta* venom. Data are presented as mean ± SEM of duplicates representative of two independent experiments. The statistical analyse was performed by One-Way ANOVA followed by Tukey HSD test. (*)Significant difference in relation to the buffer (*p* < .05). (#) Significant difference in relation to *L. laeta* venom (*p* < .05).

Of note, the accommodation region of compound **1** into the SMase D binding site was different from the other two compounds (Figure 3), suggesting it might have a distinct inhibition profile. This finding was experimentally corroborated by Lineweaver-Burk analysis data (Figure 2). The compounds **5** and **6**, though, were docked into an adjacent pocket to the substrate-binding site.

### 3.3. Compounds 1, 5 and 6 reduce SMases D binding to the human erythrocyte surface and control GPC cleavage

SMases D binding to erythrocytes is an important event in the mechanism of haemolysis. Cleavage of the external portions of the glycophorins by autologous cell membrane proteases, activated after SMase D binding, allows complement activation and lysis^10^. The three selected compounds significantly reduced the binding of the SMases D present in the whole venom to the erythrocytes membrane, the compound **5** being the most efficient inhibitor (Figure 3A). Analysis of the glycophorin C (GPC) expression on the erythrocyte surface showed that compounds **1** and **5** completely prevented the GPC cleavage induced by *L. laeta* venom (Figure 3B), while compound 6 showed a partial inhibition of GPC cleavage.

### 3.4. The novel inhibitors control keratinocytes cell death induced by *Loxosceles* venom and the production of MMPs

**Figure 4.**
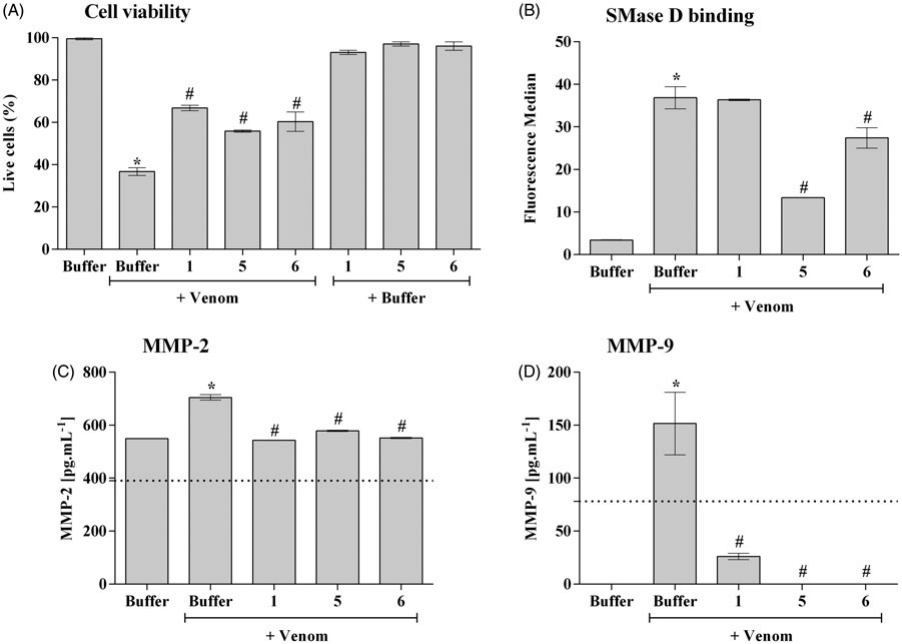
SMase D binding and cell death of human keratinocytes induced by *Loxosceles* venom. HaCaT cells (5 × 10^4^/well) were treated with 10 μg of *L. laeta* venom, preincubated or not with each inhibitor to evaluate cell death, binding of the SMase D to the keratinocyte cell membrane and secretion of MMP-2 and MMP-9. Cell death was evaluated after 72 h of incubation by the MTT method. The ability of the toxins to bind to the surface of keratinocytes was analysed by flow cytometry using a horse polyclonal serum against *L. intermedia* SMases D. Detection of MMP-2 and 9 was performed using Calbiochem MMP- 2 and 9 ELISA Kits. Data are represented as mean ± SEM of duplicates representative of three independent experiments. Statistically analysed by One-Way ANOVA followed by Tukey HSD test. (*)Significant difference in relation to the buffer (*p* < .05); (#) Significant difference in relation to the venom (*p* < .05).

SMases D from *Loxosceles* venom can bind to human keratinocytes (HaCat cells) and induce cell death^16^^,^^17^^,^^23^. In order to analyse the action of the synthetic compounds in this model, human keratinocytes were treated with *Loxosceles* venom, in the presence or absence of the inhibitors. After 72 h of incubation, compounds **5** and **6**, but not compound **1,** also significantly reduced the binding of SMases D present in *Loxosceles* venom to the cells membrane (Figure 4A). In addition, all three compounds had partially prevented the venom-induced cell death of human keratinocytes induced by the venom (Figure 4B).

Increase in the expression, secretion and activity of MMP-2 and -9 are important events inducing cell death and skin lesion formation present in the loxoscelism^16^^,^^17^^,^^23^. Figure 4 (panels C and D) shows that, as previously described, *Loxosceles* venom induced a significant increase in the release of MMP-2 and MMP-9, in the supernatant of keratinocyte cultures, which was significantly reduced to base level or near base level by all three compounds.

### 3.5. Compounds 5 and 6 inhibit the *Loxosceles* venom-induced dermonecrosis in rabbits

**Figure 5.**
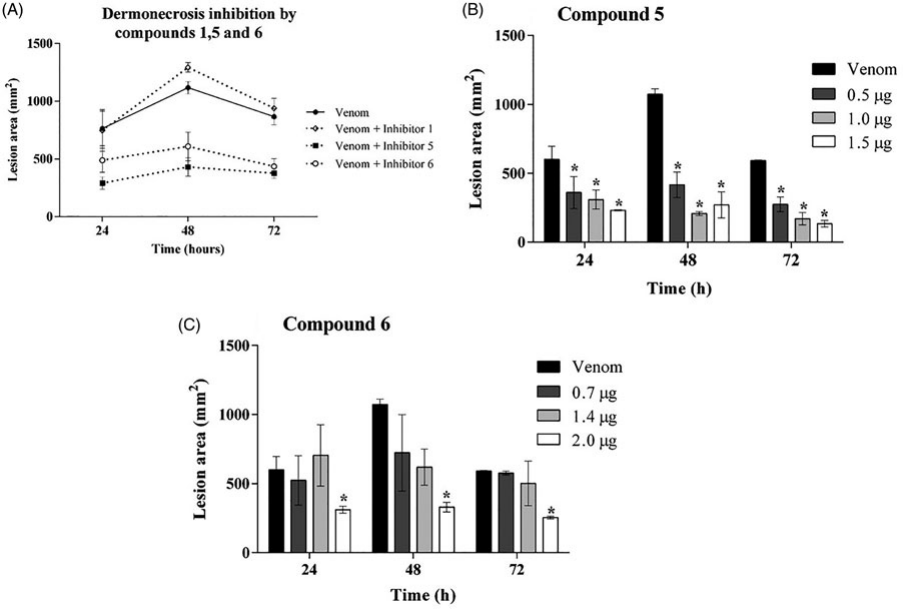
Action of inhibitors on dermonecrosis induced by *Loxosceles* venom in vivo. (**A**) Animals were inoculated intradermally with 5 μg of *L. laeta* venom in the presence or absence of inhibitors **1, 5** or **6** at the concentration of 1 µg, respectively. **(B, C)** Animals were inoculated intradermally with 5 μg of *L. laeta* venom in the presence or absence of inhibitor **5** (0.5, 1.0, 1.5 µg) or **6** (0.7, 1.4, 2.0 µg). Sterile saline solution, venom incubated with the same proportion of DMSO present in the inhibitors, only DMSO or only inhibitors were used as controls. The area of the dermonecrotic lesion was measured at 24, 48 and 72 h. Data are represented as mean ± SEM of duplicates of two independent experiments (*n* = 12 rabbits/group). Results statistically analysed by Two Way ANOVA followed by Bonferroni post-test. (*)Significant difference in relation to venom at the indicated time (*p* < .05); (**) *p* < .01; (***) *p* < .001.

The ability of the inhibitors in reducing dermonecrotic lesion, induced by *Loxosceles* venom in *in vivo* model, was evaluated by inoculating venom, in the presence or absence of the three synthetic compounds (**1**, **5** and **6),** into the skin of rabbits. 24, 48 and 72 h after inoculation. Figure 5A shows that compounds **5** and **6** reduced the lesion development by 60% and 40%, respectively, while treatment with compound **1** had no significant effect on lesion development.

Since compound **1** did not reduce the skin lesion induced by *Loxosceles* venom, a dose-response analysis was performed only with inhibitors **5** and **6**. Results revealed that, at 24, 48 and 72 h, compound **5** inhibited the venom induced lesion formation at all three used concentrations; however, no statistical differences among doses were observed (Figure 5B). Compound **6** also significantly reduced the dermocrotic lesion but only at the highest concentration used (Figure 5C).

**Figure 6.**
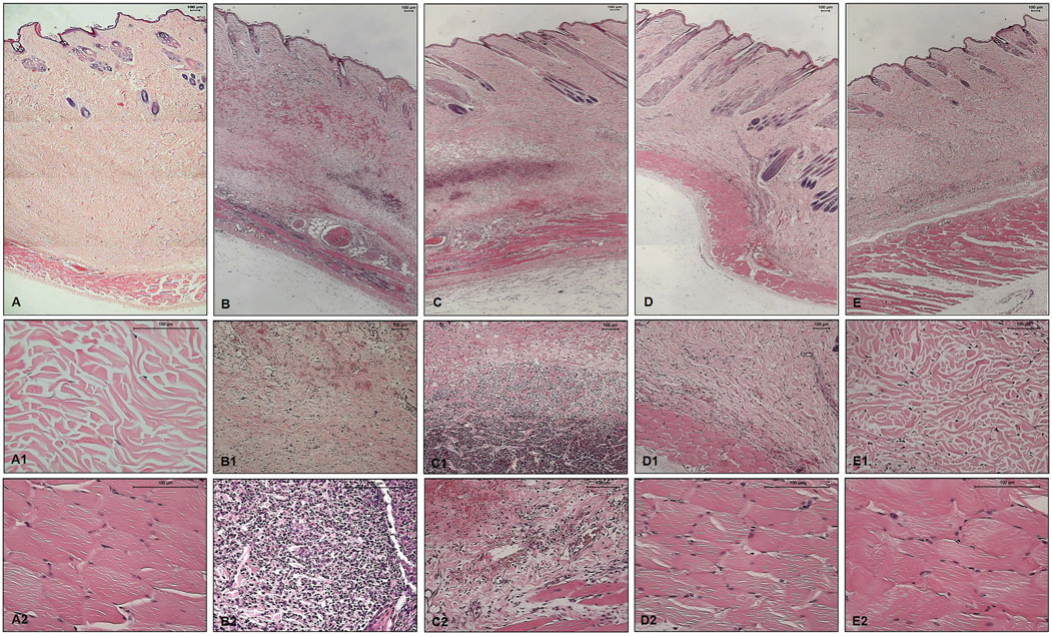
Histopathological analysis of skin fragments from rabbits inoculated with PBS, *Loxosceles* venom in the presence or absence of inhibitors 1, 5 or 6. Rabbit skin fragments inoculated with PBS, 5 μg of *L. laeta* venom in the absence or presence of inhibitors **1, 5 or 6** (1 μg), were removed 72 h after inoculation and fixed in 4% formaldehyde pH 7.4. Samples were submitted to the preparation of permanent histological slides stained with Hematoxylin-Eosin. (**A)** General appearance of the skin fragment inoculated with PBS (5× magnification). (**A1)** Detail of the collagenous area of the normal dermis (20× magnification). (**A2)** Detail of the normal adjacent muscular layer (20× magnification). (**B)** General appearance of the skin fragment inoculated with 5 μg of *L. laeta* venom in the absence of inhibitors (5× magnification). (**B1)** Detail of haemorrhagic areas and collagen disorganisation (10× magnification). (**B2)** Detail of the presence of dense inflammatory infiltrate in tissue (40× magnification). (**C)** General appearance of the skin fragment inoculated with venom in the presence of inhibitor **1** (5× magnification). (**C1)** Detail of the presence of inflammatory infiltrate and haemorrhagic areas in the tissue (10× magnification). (**C2)** Detail of the disorganisation of collagen fibres and haemorrhagic areas near the adjacent muscular layer (20× magnification). (**D)** General appearance of the skin fragment inoculated with venom in the presence of inhibitor **5** (5× magnification). (**D1)** Detail of reduced collagen disorganisation, absence of haemorrhagic areas and inflammatory infiltrate (10× magnification). (**D2)** Detail of the adjacent muscular layer (40× magnification). (**E)** General appearance of the skin fragment inoculated with venom in the presence of inhibitor **6** (5× magnification). (**E1)** Detail of reduced collagen disorganisation, absence of haemorrhagic areas and inflammatory infiltrate (20× magnification). (**E2)** Detail of the adjacent muscular layer (40× magnification). The slides were analysed and photographed using a Leica DM2500 microscope with the aid of Leica QWin Plus Y2.8 software.

Histopathological analysis of the skin fragments from rabbits inoculated with PBS (control) showed no significant alteration; nonetheless, skin fragments from rabbits inoculated with *Loxosceles* venom showed the presence of haemorrhage, dense inflammatory infiltrate, collagen disorganisation and adjacent muscle layer lesion with haemorrhage and cell infiltration (Figure 6, panels: A, A1, A2, B, B1, B2). Haemorrhagic areas were not reduced and the cell infiltrate persisted in the presence of the compound **1**. There was also a notable disorganisation of the collagen fibres and the adjacent muscular layer was injured (Figure 6 panels: C, C1, C2). The haemorrhagic areas and the cellular infiltrate were significantly reduced in the presence of compounds **5** and **6**. Collagen disorganisation was also diminished and the adjacent muscle layer was intact (Figure 6 panels: D, D1, D2, for inhibitor 5; panels: E, E1, E2, for inhibitor 6).

## 4. Discussion

Spiders belonging to the genus *Loxosceles* also known as brown spiders, have nocturnal habits, are sedentary and non-aggressive^40^. Despite being used for the capture of their prey, the venom of spiders from *Loxosceles* genus may cause human accidents, which usually occur when spiders are compressed against the human body. In these accidents the symptoms of necrosis are preceded by inflammatory signs such as oedema, thickening of the vascular endothelium, migration and accumulation of inflammatory cells, vasodilatation, intravascular coagulation, degeneration of the blood vessel wall and haemorrhage, which culminates in the formation of a darkened lesion, with confined edges, which leads to a well-defined ulcer^11^^,^^15^^,^^18^^,^^41–43^.

In accidents with *Loxosceles* spiders, a variety of therapeutic interventions like drugs such as Dapsone and Colchicine^12^, surgical debridement and skin grafts, use of corticosteroids, antibiotics and hyperbaric oxygen^44^, in addition to blood transfusion and haemodialysis^45^ have been proposed. However, a definitive and completely effective therapeutic has not been established yet. In Brazil, the most commonly used treatment for loxoscelism is the antivenom. Although some authors believe that this treatment reduces the risk of developing the systemic loxoscelism^46^, there is no consensus about its efficacy on local lesions^13^. In addition, serum therapy is not used in other countries where brown spider bites also cause a considerable number of human accidents, as USA^47–49^. Thus, the search of new potential inhibitory molecules is important for the establishment of more effective therapeutics for the loxoscelism.

Several studies have shown that the main toxic component of *Loxosceles* venoms is the sphingomyelinase D^18^ and therefore, these enzymes can be considered the most important targets for the development of more effective therapies for loxoscelism^18^. Based on the structure of SMase D from *Loxosceles laeta* and in molecular docking studies, we identified chemical compounds with potential to interact with sphingomyelinases and to inhibit their toxic activity.

Our initial *in vitro* inhibition studies showed that all 14 compounds selected by molecular docking interfered, with different intensities, with sphingomyelinase D activity (data not shown). Three compounds, belonging to the benzene sulphonate class of organic compounds, named as 1, 5, 6, induced inhibition greater than 20% and responded in a dose-dependent manner and, therefore, they were chosen to continue the study.

Among inhibitors of cellular endogenous sphingomyelinases, mechanisms of competitive and non-competitive inhibition have been identified, with variable *K*_i_ values from 1.6 to 34.2 µM^50–54^. Analysis of the inhibition mechanism of the compounds studied here showed that compounds **5** and **6** exert their inhibitory activity on SMase D in an uncompetitive manner with *K*_i_ values of 0.49 and 0.59 μM, respectively. This type of inhibition is considered a special case and occasionally observed, mainly with multi-substrate enzymes. Uncompetitive inhibitors do not necessarily resemble the substrate, and it is presumed to cause a distortion at the active site, thereby rendering the enzyme catalytically inactive without affecting the substrate binding^55^. Compound **1**, in turn, demonstrated a mixed inhibition behaviour with *K*_i_ value of 0.54 μM. It is possible for mixed inhibitors to bind to the active site or to a site other than the substrate. Despite the interesting differences between the mechanisms of inhibition between compounds **5** and **6** and compound **1**, the most relevant feature was their K*_i_* values, suggesting them to be good inhibitors, with potency ranging from 0.49 to 0.59 × 10^−7^ M.

Regarding the findings from molecular docking simulations, the formation of complex ligand-protein (calculated binding affinity) was energetically favourable to all three novel inhibitors (**1**, **5**, **6**). They established molecular interactions with the following amino acid residues in the SMase D binding site: Pro134, Ser166, Gly167, Pro168, Tyr169, Leu170, Leu173 and Thr199 (Table 3). Of note, the compounds **5** (*V* = 447.59 Å^3^; total score =  –51.65 kcal/mol) and **6** (*V* = 519.39 Å^3^; total score =  –48.23 kcal/mol) have showed distinct accommodation/orientation in the binding site when compared to compound **1** (*V* = 532.48 Å^3^; total score =  –74 kcal/mol), and the number of complementary amino acid residues was also different (12 and 15 residues for compounds **5** and **6**, respectively, against 24 residues for compound **1**). In addition, differences were observed, for the three compounds regarding the number of hydrogen bonding interactions (**1 **>** 6** > **5**) established in the binding process. The structural/molecular findings (calculated binding affinity and molecular interactions) have pointed out differences in the ligand-protein binding process that were validated, herein, through the *in vitro* assays of inhibitory activity (K*_i_* values, experimental binding affinity). Compound **1** showed, indeed, different type of inhibition (mixed type) in comparison to compounds **5** and **6** (uncompetitive type). It is noteworthy that the compounds **5** and **6** did not established any molecular interaction with the amino acid residues, which coordinate the metal ion in the catalytic site (Glu32, Asp34, Asp91).

The systemic loxoscelism involves complement-dependent haemolysis and requires the binding of SMases D to the surface of the erythrocytes. Subsequent activation of endogenous membrane bound metalloproteinases, results in glycophorin release making the cells susceptible to the lytic action of the autologous complement system^10^^,^^21^. Considering the above, we evaluated the ability of chemical compounds to interfere with both the binding of the SMases D present in the venom and the cleavage of glycophorin C. All three compounds (**1, 5** and **6**) were effective at reducing both events. Reduced binding of SMases D to the cell surface may be due to enzyme’s allosteric modification. Therefore, by preventing or reducing the binding of SMase D to the cell, the activation of membrane metalloproteinases and the removal of glycophorins will not occur, thus inhibiting the molecular mechanism involved in the haemolysis, which is crucial for the development of systemic loxoscelism.

Regarding cutaneous loxoscelism, in previous studies we observed an increase in MMP-2 and 9 expression in the rabbit skin inoculated with *Loxosceles* venom and in human keratinocytes treated with venom or SMase D, and this increase was related to cell death^17^^,^^23^. Corroborating these data, we showed here that the *L. laeta* venom was able to reduce keratinocytes viability, and that inhibitors **1**, **5** and **6** were effective in reducing venom-induced cell death. However, when the inhibitors’ ability to reduce/inhibit SMase D binding to the keratinocyte surface was assessed, only compounds **5** and **6** were able to significantly decrease this binding. Interestingly, while compound **1** inhibited SMase D binding to erythrocyte surfaces, it did not affect binding to keratinocytes. It is possible to consider that the SMase D region, involved in the interaction with the erythrocytes, which is compromised by inhibitor **1**, may be different from that used by the toxin for its interaction with keratinocytes membrane.

Studies have shown that matrix metalloproteinases, such as MMP-9, are important factors for the resolution of the lesions. However, increased levels of this molecule have been found in many types of chronic skin lesions^56^^,^^57^. Persistence of MMP-9 in inflammatory environments prevents keratinocytes from migrating to the newly synthesised basement membrane, making it difficult to re-epithelialize and heal lesions^58^. Likewise, MMP-2 and 9^16^^,^^17^ are involved in the development of cutaneous loxoscelism and, thus, we investigated the action of SMase D inhibitors on the production of MMPs by keratinocytes. The results presented here show that *L. laeta* venom induced a significant increase of MMP-2 and 9 and co-incubation with inhibitors **1**, **5** and **6** reduced the increase of these metalloproteases induced by the venom.

In most cases of loxoscelism, a chronic ulcer with extensive tissue destruction is formed, which can take many months to heal^11^^,^^43^. In the present study, inoculation of 5 μg of *L. laeta* venom into the rabbit dorsum produced a lesion similar to that described previously and, by measuring these lesions in the presence or absence of inhibitors, the efficacy of these compounds was evaluated. Only compounds **5** and **6** significantly reduced the lesion and its progression during 72 h. The lack of inhibition by compound **1** of the toxin interaction with keratinocytes surface as observed here may explain why compound **1** was ineffective at inhibiting the rabbit skin lesions, suggesting that the most important step for cutaneous lesions development seems to be the toxin binding to cell surfaces.

In agreement with previous studies^16^, the histopathological analysis of rabbits skin sections inoculated with *Loxosceles* venom, as conducted in this study, showed disorganisation of the collagen fibres in the dermis, dense inflammatory infiltrate, haemorrhagic areas and lesions in the adjacent muscular layer. As we showed above, inhibitors **5** and **6** reduced the dermonecrotic lesion caused by the venom and, as expected, both inhibitors reduced the disorganisation of the collagen fibres and inflammatory infiltrate. Haemorrhagic areas and muscle layer lesions were absent, indicating, again, the efficacy of inhibitors **5** and **6** in the reduction of the skin lesion caused by loxoscelism process.

The analysis of SMase D structure, resolved in the presence or not of sulphate^19^^,^^59^, allowed the definition of the acid-base catalytic mechanism involving two histidine residues, His12 and His47, as well as the role of the Mg^2+^ ion. In addition to the histidine residues, the amino acid residues Glu32, Asp34, Asp52, Asp91, Trp230, Asp233 and Asn252, are fully conserved in the *Loxosceles* species SMases D isoforms. Lys93, located in the catalytic pocket, is also highly conserved and may play a crucial role in balancing the charge during catalysis or in orienting the bound substrate.

Compound **1** was accommodated nearest by the catalytic pocket than compounds **5** and **6**. However, it interacts with only three (Asp91, Lys93, Trp230) out the nine amino acid residues located in the SMase D active site. This inhibitor was not able to abolish neither the SMase D keratinocyte surface binding nor the dermonecrosis induced by the venom. Despite not being oriented as close as compound **1** to the catalytic site, compound **6** establishes molecular interactions with Lys93 and, more importantly, with His47 (related to sphingomyelin catalysis by SMase D) in the catalytic pocket. Comparing the complementary residues in the binding pocket found for the three compounds, we could hypothesise that Asn200 and Phe201 are important for the SMase D interaction with keratinocyte surfaces, and the subsequent dermonecrosis development, since they did not participate in the compound **1** binding process. Another possibility would be related to the binding region in which the compounds **5** and **6** were docked. That binding site, adjacent to the substrate-binding region, could be important for the recognition process between SMase D and keratinocytes surfaces.

## 5. Conclusions

In summary, the results obtained indicate that even not being the molecules that received the highest Goldscore values in the docking studies, compounds **5** and **6** were effective in modulating the activity of SMase D from *Loxosceles* venom in all tested aspects. Due to the need to develop effective treatments to treat loxoscelism, complementary to serum therapy, these compounds presented here may have therapeutic potential and can be used as prototype for the development of new complementary drugs in the treatment of envenomation by *Loxosceles* spiders.

## Supplementary Material

Supplemental Material
