# Supplementary material for: Targeting Loxosceles spider Sphingomyelinase D with small-molecule inhibitors as a potential therapeutic approach for loxoscelism
Source: J Enzyme Inhib Med Chem. 2019 Jan 7;34(1):310–21. doi: 10.1080/14756366.2018.1546698 (PMC6327989; doi:10.1080/14756366.2018.1546698)
Supplement: Supplemental Material [file IENZ_A_1546698_SM5267.pdf]

Compound 2

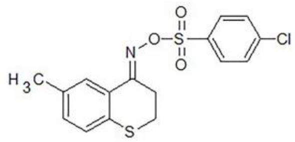

Compound 3

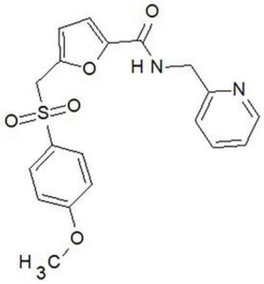

Compound 4

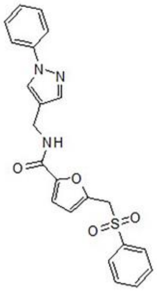

Compound 7

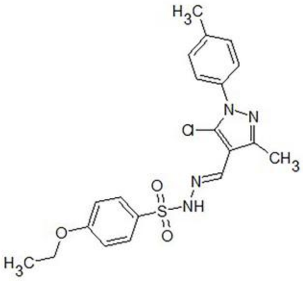

Compound 8

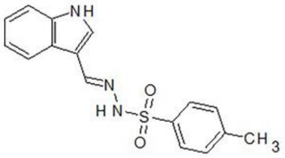

Compound 9

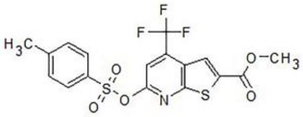

Compound 10

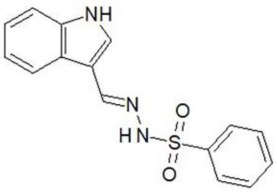

Compound 11

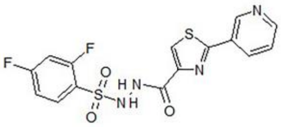

Compound 12

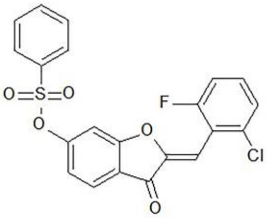

Compound 13

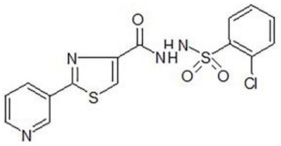

Compound 14

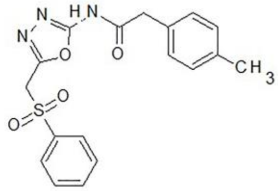

Supplementary Figure 1
